# Toward developing recombinant gonadotropin-based hormone therapies for increasing fertility in the flatfish Senegalese sole

**DOI:** 10.1371/journal.pone.0174387

**Published:** 2017-03-22

**Authors:** François Chauvigné, Judith Ollé, Wendy González, Neil Duncan, Ignacio Giménez, Joan Cerdà

**Affiliations:** 1 Institut de Recerca i Tecnologia Agroalimentàries (IRTA)-Institut de Ciències del Mar, Consejo Superior de Investigaciones Científicas (CSIC), Barcelona, Spain; 2 IRTA, Sant Carles de la Ràpita, Tarragona, Spain; 3 Rara Avis Biotec, S. L., Valencia, Spain; Universitat de Barcelona, SPAIN

## Abstract

Captive flatfishes, such as the Senegalese sole, typically produce very low volumes of sperm. This situation is particularly prevalent in the first generation (F1) of reared sole males, which limits the development of artificial fertilization methods and the implementation of selective breeding programs. In this study, we investigated whether combined treatments with homologous recombinant follicle-stimulating (rFsh) and luteinizing (rLh) hormones, produced in a mammalian host system, could stimulate spermatogenesis and enhance sperm production in Senegalese sole F1 males. In an initial autumn/winter experiment, weekly intramuscular injections with increasing doses of rFsh over 9 weeks resulted in the stimulation of gonad weight, androgen release, germ cell proliferation and entry into meiosis, and the expression of different spermatogenesis-related genes, whereas a subsequent single rLh injection potentiated spermatozoa differentiation. In a second late winter/spring trial corresponding to the sole’s natural prespawning and spawning periods, we tested the effect of repeated rLh injections on the amount and quality of sperm produced by males previously treated with rFsh for 4, 6, 8 or 10 weeks. These latter results showed that the combination of rFsh and rLh treatments could increase sperm production up to 7 times, and slightly improve the motility of the spermatozoa, although a high variability in the response was found. However, sustained administration of rFsh during spawning markedly diminished Leydig cell survival and the steroidogenic potential of the testis. These data suggest that *in vivo* application of rFsh and rLh is effective at stimulating spermatogenesis and sperm production in Senegalese sole F1 males, setting the basis for the future establishment of recombinant gonadotropin-based hormone therapies to ameliorate reproductive dysfunctions of this species.

## Introduction

Reproductive dysfunctions of male fish are often encountered in the aquaculture of flatfishes (Pleuronectiformes), a group of fishes of high commercial value worldwide [1–5]. The Senegalese sole (*Solea senegalensis*) is a highly prized flatfish with growing commercial importance in Southern Europe and Asia. However, the mass production of alevins of this species largely relies on wild male breeders acclimated to captivity, since the first generation (F1) of cultured males usually show low or more often zero natural fertilization rates [4, 6–8]. This situation hampers the selective breeding of farmed populations and makes the industry unsustainable in the long term. The development of *in vitro* fertilization methods has been envisaged as an alternative approach, but the low quantity (<130 μl) and variable quality of the sperm that the sole males typically produce make these methods impractical at an industrial level [7, 9–13]. The use of hormone therapies to enhance sperm production could provide a solution to ameliorate the reproductive dysfunctions arising in cultured fish.

Compared to other marine teleosts with highly cyclic (seasonal) testicular development, the Senegalese sole shows a more complex spermatogenic process. In this species, germ cell development is semicystic and asynchronous, with spermatogenesis occurring almost all year-round from the first sexual maturation, and spermiogenesis (spermatozoa differentiation) taking place from spermatids released into the seminiferous lobules [14, 15]. As in mammals, spermatogenesis in the Senegalese sole might be regulated by the pituitary gonadotropins follicle-stimulating (Fsh) and luteinizing (Lh) hormones through the binding to their cognate receptors in the testis, the Fsh receptor (Fshr) and the Lh/chorionic gonadotropin receptor (Lhcgr) [16, 17]. For the investigation of the gonadotropic regulation of testis development in sole, biologically active homologous single-chain recombinant gonadotropins (rFsh and rLh) have recently been produced in Chinese hamster ovary (CHO) cells, which can stimulate the synthesis and release of the major androgen 11-ketotestosterone (11-KT) both *in vitro* and *in vivo* [18, 19]. The studies *in vitro* suggest that Fsh is primarily involved in early spermatogenesis through the activation of the Fshra in both the Sertoli and the steroidogenic Leydig cells, whereas Lh preferentially regulates the later stages of germ cell development and the differentiation of spermatozoa through the Lhcgrba present in Leydig cells as well as in spermatids [18, 20]. Therefore, direct activation of the Lhcgrba in haploid spermatids might represent a key regulatory mechanism for the control of spermiogenesis in Senegalese sole [20].

Studies in wild and F1 sole males, using newly developed enzyme-linked immunosorbent assays (ELISAs) specific for Fsh and Lh [21, 22], have revealed lower levels of plasma Lh in F1 males than in wild males, while the levels of Fsh and androgens are comparable, suggesting that the low fertilization capability of F1 males may be related to low circulating levels of Lh [22]. Based on these observations it seems plausible that hormone therapies using the administration of the hypothalamic gonadotropin-releasing hormone analogue (GnRHa), which can stimulate the pituitary release of endogenous gonadotropins, might stimulate sperm production in sole as in other farmed teleosts [3]. However, perhaps as a consequence of the intricate type of spermatogenesis in Senegalese sole, GnRHa treatments do not significantly enhance sperm production, although they are effective at promoting ovulation in females [6, 23, 24]. Thus, GnRHa treatment, with or without precursors of 11-KT, such as 11-ketoandrostenedione, or dopaminergic inhibitors, do not result in a major increase in sperm volume or density, although it may induce a transient elevation of circulating androgens [6, 7, 11, 25], increase sperm motility [7], or increment the hydration of the sperm [23] as seen in other flatfishes [26–28]. To date, the most efficient treatment for improving the fertilization potential of sole F1 males has been the use of human chorionic gonadotropin (hCG), a hormone closely related to the mammalian Lh [29], which directly targets the gonad and can increase the androgen plasma levels and slightly increase the fertilization rates [24, 30]. However, these studies have not analyzed in detail the effect of hCG on the progression of spermatogenesis, and have not assessed the quantity and quality of the sperm produced. In addition, the latency and the optimal doses of hCG to promote spermiation are not known. Such parameters are critical to the successful establishment of artificial fertilization protocols.

The studies in Senegalese sole, as well as in other teleosts [31–38], suggest that the use of recombinant gonadotropins could be an effective method to enhance sperm production and quality in cultured fish. In addition to CHO cells, teleost recombinant gonadotropins have been produced in different heterologous eukaryotic systems, such as the yeast *Pichia pastoris* [31, 32, 36, 39], the baculovirus-silkworm system [33, 37] and insect Sf9 cells [34]. However, the type of glycosylation of the hormones that occurs in mammalian systems renders the polypeptides more stable in fish plasma than those produced in other heterologous hosts [38], and can also enhance their biological activity [40, 41]. Homologous rFsh and rLh can also be more advantageous than the hCG, since the hCG is a high molecular mass hormone with a long half-life in the circulation, which can cause immune responses in the animal after repeated or prolonged exposure, resulting in the loss of effectiveness at medium and long term [42]. In addition, due to the cross activation of the Senegalese sole Fshra by hCG [17, 18], it is possible that by continuously supplying this hormone both the Fshra and Lhcgrba are activated, which in the asynchronous testis of the sole may trigger concomitant stimulatory and inhibitory mechanisms that can subsequently negatively affect the processes of spermatogenesis and/or sperm maturation.

In the present study, we report the first attempts to develop rFsh- and rLh-based hormone therapies to promote spermatogenesis and enhance sperm production in Senegalese sole F1 males. Our data show that treatment with rFsh is effective for inducing testicular growth and for stimulating germ cell development, and that combined treatments with rFsh and rLh can improve the amount of sperm produced.

## Materials and methods

### Fish and sampling procedures

Approximately two-year old adult Senegalese sole males, F1 offspring of wild captive fish, were obtained from the commercial company Stolt Sea Farm S.A. (Spain), and transported to the IRTA fish research facilities at Sant Carles de la Ràpita (Spain). Fish were held in fiber glass tanks of 10 m^3^ connected to a recirculation system (IRTAmar^®^), and were fed five days a week with 0.75% of wet feed (mussels and polychaetes) and 0.55% of dry feed (balance diet) of the total biomass. Fish were maintained during and for at least 3 months before the experiments under natural photoperiod and controlled simulated natural temperature.

For the samplings, fish were sedated with 60 mg/l tricaine methanesulfonate (MS-222; Sigma-Aldrich),weighed, and a sample of 0.5–1 ml of blood collected from the caudal vein using a syringe previously coated with 0.5 M EDTA pH 8. The blood was placed into a tube containing 5 μl EDTA, centrifuged at 3000 x g for 15 min at 4°C, and the plasma aliquoted and stored at -80°C. In some experiments, fish were immediately sacrificed by decapitation, and the testes removed in order to determine the gonadosomatic index (GSI; testes weight/fish weight x 100). The right testis was entirely processed for histology or cut into two pieces and processed separately for histology and immunohistochemistry. The periphery of the left testis, mostly containing the cortical region, was cut into small pieces (~20 mg), deep-frozen in liquid nitrogen, and stored at -80°C. The sperm was collected from anaesthetized fish by applying soft pressure and gentle massage to the abdominal area where the testes are situated and further along the sperm ducts to the cloaca. The sperm was collected with a micro haematocrit 75 x 1.15 mm capillary (Brand GMBH) in order to estimate the volume of the ejaculate as accurately as possible. The sperm was immediately transferred into a 1.5-ml tube at room temperature (~20°C) and the motility evaluated within 30 min.

The procedures relating to the care and use of animals and sample collection were conducted in accordance with the protocols approved by the Ethics Committee (EC) of the Institut de Recerca i Tecnología Agroalimentàries (IRTA) following the European Union Council Guidelines (86/609/EU). The present study was also specifically approved by IRTA EC.

### Antibodies and reagents

The mouse monoclonal antibodies against 5-bromo-2'-deoxyuridine (BrdU) and rat proliferating cell nuclear antigen (PCNA) were purchased from Developmental Studies Hybridoma Bank (G3G4; University of Iowa) and Genetex Inc. (GTX-20029), respectively, the rabbit polyclonal antibody against cleaved caspase-3 (Casp3 Asp175) was acquired from Cell Signaling Technology, Inc. (9661), and the sheep polyclonal anti-digoxygenin (DIG) antibody was from Roche Applied Science (11093274910). The Alexa Fluor 647-conjugated wheat germ agglutinin (WGA) was obtained from Life Technologies Corp. (W32466). The specific antibodies against Senegalese sole Fsh β and Lh β subunits have been previously characterized [21, 22]. The secondary antibodies employed included Alexa Fluor 488-coupled goat anti-rabbit and anti-mouse IgG (A-11008 and A-11029, respectively; Life Technologies Corp.), and horseradish peroxidase-coupled goat anti rabbit IgG (sc-2004; Santa Cruz Biotechnology, Inc.). All other reagents and kits were from Life Technologies Corp. unless stated otherwise.

### Production of Senegalese sole recombinant gonadotropins and biological half-life

Single-chain Senegalese sole rFsh and rLh were produced by Rara Avis Biotec (Valencia, Spain) essentially as described previously [18]. In brief, CHO cells were transfected with expression constructs encoding fusion proteins containing the entire coding sequence of Senegalese sole Fshβ (GenBank accession no. ABW81403) or Lhβ subunit (GenBank accession no. ABW81404), a carboxyl-terminal peptide-like sequence of hCG β subunit as a linker to assist the chimerization of the subunits, and the mature sequence of the Senegalese sole glycoprotein hormone α subunit (Cga; GenBank accession no. ABW81405). The cells were cultured in suspension for 120 h, and the secreted recombinant hormones were subsequently purified from the culture medium by ion exchange chromatography. The hormones were concentrated, and quantified by semiquantitative Western blot, using an antibody against European seabass (*Dicentrarchus labrax*) Cga (gently provided by A. Gómez, CSIC, Spain), as well as by specific enzyme-linked immunosorbent assay (ELISA) as described previously [21, 22]. Hormones were stored at -80°C and diluted just before use with saline (0.9% NaCl) to various concentrations (2.5–10 μg/ml) which enabled all treatment groups to be injected with the same volume containing the appropriate hormone dose.

The glycosylation of the hormones was confirmed by sodium dodecyl sulfate polyacrylamide gel electrophoresis (SDS-PAGE) followed by immunoblotting. For this, recombinant gonadotropins (0.5 μg) were diluted in Laemmli sample buffer containing SDS and DTT, denaturated at 95°C, and separated in 12% acrylamide gels. The glycosylation state of the proteins was assessed by incubating the samples with 500 units of N-Glycosidase F (PNGase F; New England Biolabs Inc.) for 2 h at 37°C prior to electrophoresis. Proteins were blotted onto Immun-Blot^®^ nitrocellulose membranes (Bio-Rad, Spain), blocked in 5% nonfat dry milk in TBST (20 mM Tris, 140 mM NaCl, 0.1% Tween, pH 8) and incubated overnight at 4°C with the Senegalese sole Fshβ and Lhβ antisera diluted 1:5000 in blocking buffer. Bound antibodies were detected with horseradish peroxidase-coupled goat anti rabbit IgG secondary antibody (1:10000) and Western HRP substrate (Millipore).

To determine the plasma half-life of the hormones, male and female sole (347 ± 7 g) maintained at 16 ± 1°C and natural photoperiod were injected intramuscularly with saline or 6 μg/fish of rFsh or rLh (17.1 ± 0.4 μg/kg; *n* = 5 per group), and blood samples were collected just before injection and at 1, 3, 7, 14 and 21 days after injection. Plasma levels of endogenous and recombinant gonadotropins were determined by ELISA as described above.

### Experimental setup

**Fig 1:**
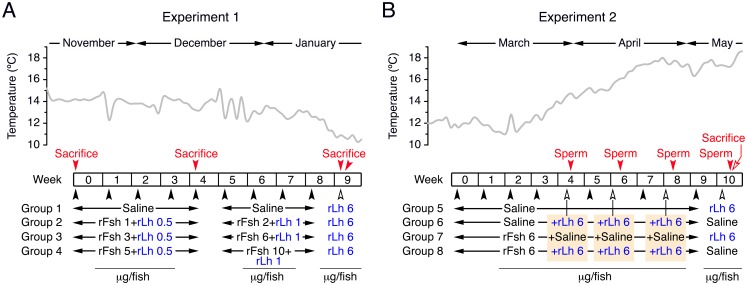
Schematic representation of the experimental setup. (A) In experiment 1, carried out in autumn and winter, the effect of different doses of rFsh (1–2, 3–6 or 5–10 μg/fish) plus a low rLh dose (0.5–1 μg/fish) during 4 or 9 weeks on spermatogenesis was tested. Control fish (Group 1) was treated with saline. At week 9, all groups were treated with a high dose of rLh (6 μg/fish). (B) In experiment 2, carried out during late winter and spring, the effect of repeated treatment with rLh (6 μg/fish) on sperm production by males previously treated with saline or rFsh (6 μg/fish) during 4, 6 and 8 weeks was tested. After 10 weeks of saline or rFsh treatment, the rLh induction was switched between the groups. In both experiments, the black arrowheads indicate the injection days, and the red arrowheads the type of sampling (sacrifice or sperm collection) during the trials. The temperature of the holding tanks during each experiment is indicated in the upper panels. See other details in Materials and methods.

The designs of the two experiments carried out in this study to investigate the effect of rFsh and rLh on spermatogenesis and sperm production are depicted in Fig 1.

#### Experiment 1

This experiment was performed over 9 weeks from November to January to investigate the effect of different rFsh doses plus a low rLh dose during different times on germ cell development (Fig 1A). During the first 4 weeks, four groups of males (426 ± 16 g; *n* = 13) were injected intramuscularly once a week with saline (Group 1) or three doses of rFsh, 1, 3 or 5 μg/fish (2.9 ± 0.5, 5.7 ± 0.7 or 12.1 ± 1.7 μg/kg; Groups 2, 3 and 4, respectively), in combination with a low dose of 0.5 μg/fish rLh (1.3 ± 0.1 μg/kg). During the following 4 weeks, Groups 2 to 4 were treated with the double of the rFsh doses, 2, 6 or 10 μg/fish (5.0 ± 0.7, 15.5 ± 2.0 or 24.1 ± 4.7 μg/kg), plus 1 μg/fish rLh (2.5 ± 0.2 μg/kg). Before the first injection (time zero), and after 4 and 9 weeks of treatment a subsample of fish (*n* = 5) was sacrificed. At week 9, the remaining fish in each group (*n* = 3) were treated with 6 μg/fish of rLh (13.7 ± 1.4 μg/kg) and sacrificed 24 h later.

#### Experiment 2

In this trial, we tested the effect of rLh, previous treatment or not with rFsh for different times, on sperm production (Fig 1B). This experiment was conducted for 10 weeks from March to May, thus spanning the natural breeding season of Senegalese sole in spring, when spermiation is slightly enhanced during the annual fluctuation of female ovulation [15]. Fourty males (354 ± 7 g) were divided into four groups (*n* = 10) and were injected intramuscularly with saline (Groups 5 and 6) or 6 μg/fish rFsh (16.2 ± 0.3 μg/kg; Groups 7 and 8) once a week during 9 weeks. At week 4, 6 and 8, fish from Groups 6 and 8 were injected in addition with 6 μg/fish rLh (16.6 ± 0.3 μg/kg), whereas Groups 5 and 7 were treated with saline. Sperm and blood samples were collected 24–48 h after each rLh injection. At week 10, the treatments were switched, Groups 5 and 7 received 6 μg/fish rLh (17.0 ± 0.5 μg/kg) and Groups 6 and 8 were treated with saline. Sperm and blood samples were also collected 48 h after these last injections, and 5 fish per group were sacrificed the following day (72 h).

### Steroid determination

Levels of 11-KT in plasma were determined by commercial enzyme immunosorbent assay (EIA; Cayman Chemical Company) as described previously [18, 22]. Free steroids were extracted from plasma (3.5 μl) in methanol and the resulting pellet was diluted 1:150 in EIA buffer (0.1 M K_2_HPO_4_/KH_2_PO_4_, 1.54 mM sodium azide, 0.4 M NaCl, 1 mM EDTA, and 0.1% BSA, pH 7.4). All samples were analyzed in duplicate, and a separate standard curve was run for each EIA plate.

### Histological analysis

Testis pieces were fixed in Bouin’s fluid for 16 h at room temperature, dehydrated and embedded in paraplast (Sigma-Aldrich). The testis biopsies from all treatment groups were oriented in the molds in the same manner to obtain sagital sections of the same testicular area. Sections of 7 μm in thickness were further attached to UltraStick/UltraFrost Adhesion slides (Electron Microscopy Sciences) and stained with hematoxylin and eosin as previously described [18].

Somatic (Sertoli and Leydig cells) and germ cells were identified following the criteria by García-López et al. [14]. The area of the seminiferous tubules and the number of Leydig cells and each type of germ cell in the cortical and medullar regions of the testis were assessed using the NIS-element AR 4.30.02 software (Nikon). The number of spermatogonia (type A and B), spermatocytes, spermatids and spermatozoa was scored in 10 tubules of 5–10 different areas of the cortex and medulla for each fish and normalized by the area of the whole tubule. Similarly, the number of Leydig cells was counted in six foci in 5–10 different areas of the cortex and medulla per fish. To estimate the number of spermatozoa in the lumen of the sperm duct, the spermatozoa were counted in nine different areas of 0.22 mm^2^ of the lumen of the duct per fish.

### Immunofluorescence microscopy

Testis samples were incubated with 10 μM BrdU for 1 h in L-15 without phenol red supplemented with 10 mM HEPES, 0.5% bovine serum albumin (Sigma-Aldrich), 0.4 mg/ml Fungizone (Sigma-Aldrich) and 200 ug/ml penicillin/streptomycin, washed twice in L-15 and fixed in 4% paraformaldehyde (PFA; Sigma-Aldrich) for 6 h at room temperature. After washing, dehydration, and embedding in paraplast, sections of 7 μm thickness were attached to UltraStick/UltraFrost Adhesion slides and rehydrated before permeabilization. For the BrdU antibody, sections were permeabilized with boiling citrate at 0.01 M and pH 6 for 5 min, repeated 3 times. After the citrate solution was cooled down at room temperature, the slides were washed in phosphate buffer solution (PBS; 20 mM Na_3_PO_4_, 500 mM NaCl, pH 7.4) and subjected to a second permeabilization step in 2 N HCl for 20 min at 37°C, then quickly washed in PBS and exposed to 0.07 M NaOH for 10 min. Sections were rinsed again in PBS and incubated with 0.2% Triton X-100 for 15 min at room temperature. For the Casp-3 antibody, the permeabilization was carried out in 0.01 M citrate for 30 min. After permeabilization, sections were blocked in 5% goat serum and 0.1% BSA in PBS with 0.1% Tween-20 (PBST) for 1 h before incubation with the anti-BrdU (1:600) or Casp-3 (1:200) antibodies overnight at 4°C. Adjacent sections were incubated without antibody as a negative control. After washing in PBS, sections were exposed to an Alexa Fluor 488-coupled anti-rabbit IgG secondary antibody (1:1000) for 1 h at room temperature. The nuclei and membranes/extracellular matrix were counterstained (1:3000) with 4’,6-diamidino-2-phenylindole (DAPI; Sigma-Aldrich) and Alexa Fluor 647-conjugated WGA, respectively. The slides were mounted with fluoromount aqueous anti-fading medium (Sigma-Aldrich) and immunoreactions were photographed with a Zeiss Axio Imager Z1/ApoTome fluorescence microscope (Carl Zeiss Corp.). Images from each treatment group were taken with the same fluorescence intensity and exposure.

The BrdU and PCNA labeling (mitotic) indices were calculated as the percentage of marker-positive cells (spermatogonia, Sertoli cells or Leydig cells) with respect to the total number of cells in each tubule, or in one foci for Leydig cells, scored for at least 5 tubules in 5 different testicular areas from cortex and medulla per fish. The percentage of Casp-3-expressing Leydig cells per foci was determined in the same way.

### Fluorescent In Situ Hybridization (FISH)

Expression of 17β-hydroxysteroid dehydrogenase (*hsd17b*) in the testis, as a marker of Leydig cells, was carried out by FISH using specific DIG-labeled *hsd17b* riboprobes [19] following previously described methods [18, 43]. In this case, the green fluorescence was obtained by using Alexa Fluor 488-conjugated goat anti-mouse IgG secondary antibodies (1:500) in TBST for 2 h at room temperature. Sections were washed in PBS, counterstained with WGA and DAPI, and mounted as above.

### RNA extraction and real-time quantitative PCR

Extraction of total RNA from testis was carried out using the GenElute Mammalian Total RNA Kit (Sigma-Aldrich). Samples were treated with On-column DNAse I Digestion Kit (Sigma-Aldrich), and 0.5 μg of total RNA was reverse transcribed using 0.5 μg oligo(dT)_17_, 1 mM dNTPs, 40 IU RNAse inhibitor, and 10 IU SuperScript^®^ II Reverse Transcriptase enzyme for 1.5 h at 42°C. Real-time quantitative RT-PCR (qRT-PCR) was carried out using 5 μl of SYBR Green qPCR master mix, 1 μl of the cDNA 1:10 diluted, and 0.5 μM of each forward and reverse primers for *fshra*, *lhcgrba*, Piwi-like protein 2 (*piwil2*), structural maintenance of chromosomes protein form b (*smc1b*), septin 7a (*sep7a*), radial spoke head 1 homolog (*rsph1*), *hsd17b*, and 11β-hydroxysteroid dehydrogenase type 2 (*hsd11b2*). Amounts of the target transcripts were normalized to those of the elongation factor *ef1a1*. The sequence of the specific primers for all the genes has been previously described [19], and their efficiency was estimated by the generation of a standard curve for each primer pair from 10-fold serial dilutions (from 10 to 0.0001) of a pool of mixed samples from testes at time 0. For the assays, each sample was determined in duplicate on 384-well plates using the ABI PRISM 7900HT sequence detection system. The amplification protocol was an initial denaturation and activation step at 50°C for 2 min and 95°C for 10 min, followed by 40 cycles of 95°C for 15 s and 63°C for 1 min and finally a temperature-determining dissociation step was carried out at 95°C for 15 s, 60°C for 15 s, and 95°C for 15 s. The standard curves exhibited a correlation coefficient >0.99 and efficiency of ~2. Changes in gene expression were determined as fold-changes with respect to samples collected at time zero using the 2^-ΔΔCt^ method (Experiment 1), or as relative quantities calculated from the standard curves (Experiment 2).

### Activation of sperm motility and evaluation of sperm motion kinetics

An aliquot of freshly collected sperm was diluted 1:10 with non-activating medium (NAM; in mM: 59.89 NaCl, 1.48 KCl, 12.92 MgCl_2_, 3.51 CaCl_2_, 20 NaHCO_3_ and 10 mg/ml BSA, pH 7.7; 310 mOsm) [44]. The concentration of spermatozoa was evaluated using a computer assisted sperm analysis (CASA) system (ISASv1 software, Proiser, Spain) coupled to a phase contrast microscope (Nikon Eclipse 50i, Nikon) equipped with a x10 negative phase contrast objective. Sperm counts were done in three different regions of the sperm counting chamber to avoid miscalculations. The total amount of spermatozoa per ejaculate was then normalized by the weight of the fish. Sperm samples were subsequently diluted in NAM at a concentration of 10^9^ cells/ml. Motility was measured immediately after 1:6 dilution in artificial sea water (ASW; in mM: 420.9 NaCl, 9 KCl, 22.9 MgCl_2_, 25.5 MgSO_4_, 9.25 CaCl_2_, 2.1 NaHCO_3_, pH 8.0; 1100 mOsm) [45]. Samples with low sperm concentration were used directly for activation. The sperm kinetic parameters, the percentage of total motile and progressive sperm, and the curvilinear velocity (i.e the average velocity measured over the actual point to point track followed by the sperm cell; VCL, μm/s), were recorded by CASA just after activation in filtered ASW (5 sec) and every min until motility completely stopped. The measurements were carried out in duplicate or triplicate for each ejaculate.

### Statistical analysis

Results are expressed as the means ± SEM. Comparisons between groups were made by one- or two-way ANOVA, after log or arcsine transformation of the data when needed. If significant differences were detected (*P* < 0.05) means were compared by the Duncan’s multiple range test. Statistical and regression analyses were carried out using the Statgraphics Plus 4.1 software (Statistical Graphics Corp., USA).

## Results

### Biological half-life of Senegalese sole rFsh and rLh

**Fig 2:**
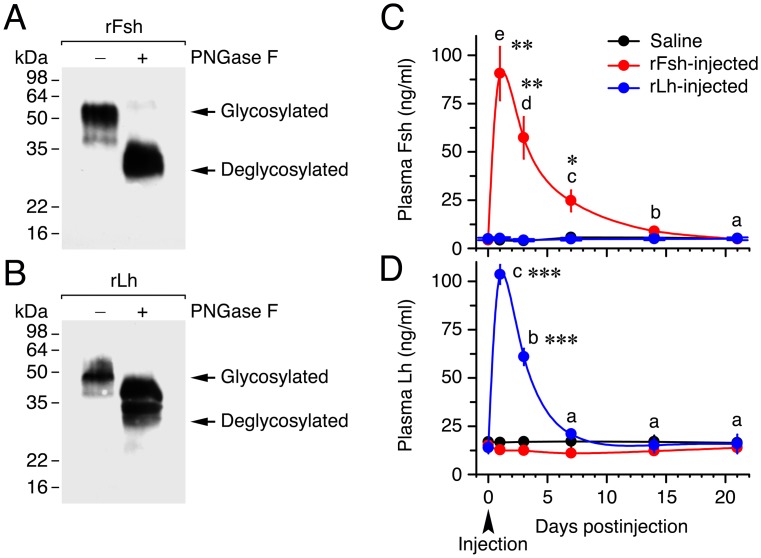
Characterization of Senegalese sole recombinant gonadotropins and half-life of the hormones in plasma. (A, B) Characterization of CHO cells-expressed recombinant single-chain Fsh and Lh (rFsh and rLh) by Western blot analysis and deglycosylation. Concentrated supernatants of cell cultures were separated by 12% SDS-PAGE and immunoreacted with antibodies against Senegalese sole Fsh and Lh β subunits. For deglycosylation, denatured and reduced proteins were incubated with (+) or without (-) N-glycosidase F (PNGase F). Molecular mass markers (kDa) are on the left. (C, D) Time-course of plasma Fsh and Lh concentrations in fish (mean ± SEM; *n* = 5 fish) after intramuscular injection of saline or 6 μg/fish (17.5 ± 0.6 μg/kg) of rFsh or rLh. *, *P* < 0.05; **, *P* < 0.01; **, *P* < 0.001; with respect to the saline-injected fish.

The glycosylation of the rFsh and rLh produced in CHO cells was confirmed by SDS-PAGE and Western blotting using specific antibodies against Senegalese sole Fshβ and Lhβ previously developed [21, 22] (Fig 2). As expected, each sole Fsh and Lh antiserum was able to detect one thick band, corresponding to rFsh (Fig 2A) and rLh (Fig 2B), respectively. Both immunoreactive polypeptides appeared to be extensively glycosylated by asparagine-linked glycans since treatment with PNGase F reduced their apparent molecular mass from ~50 kDa to ~35 kDa, although PNGase F-mediated deglycosylation seemed to be more efficient for rFsh than for rLh.

To investigate the half-life of the recombinant hormones in sole plasma, fish were injected once with 6 μg/fish of rFsh or rLh, or saline (control), and the plasma levels of Fsh and Lh determined in the three groups at different times during 21 days. Before rFsh injection, the circulating levels of Fsh were 4 ng/ml, and one day after rFsh injection these levels reached 91 ng/ml (Fig 2C). Plasma Fsh levels were subsequently reduced by 39% and 77% at 3 and 7 days, respectively, and returned to basal levels after 14 days. The Lh plasma levels before rLh injection were of 14 ng/ml, and these increased to 101 ng/ml 1 day after rLh treatment (Fig 2D). The plasma levels of Lh then dropped by 48% after 3 days, and these levels were no longer different than those of the control fish 7 days after injection. These data indicate that the half-life of rFsh and rLh was of ~5 and ~3 days, respectively, and that injection with relatively high doses of rFsh or rLh do not affect, respectively, the endogenous levels of Lh or Fsh (Fig 2C and 2D).

### Experiment 1: effect of rFsh on spermatogenesis

The first trial was aimed at examining the effect of a weekly injection with three rFsh doses (1–2, 3–6 and 5–10 μg/fish, for Groups 2, 3 and 4, respectively) during two time periods (4 and 9 weeks) on germ cell development. The rFsh doses and the injection frequency were established based on the previously determined half-life of plasma rFsh to maintain high levels of circulating hormone during the entire experimental period. The rFsh treatments were combined with a low dose of rLh (0.5–1 μg/fish), since previous experiments on sole F1 males have detected low levels of endogenous circulating Lh (1–5 ng/ml) during winter and before the spawning in spring [22]. At the end of this experiment, we also tested whether the longest rFsh treatment could have a positive effect on spermiogenesis by treating saline- and rFsh-injected males with rLh (6 μg/fish).

#### Treatment with rFsh and rLh stimulate testis growth and androgen release

**Fig 3:**
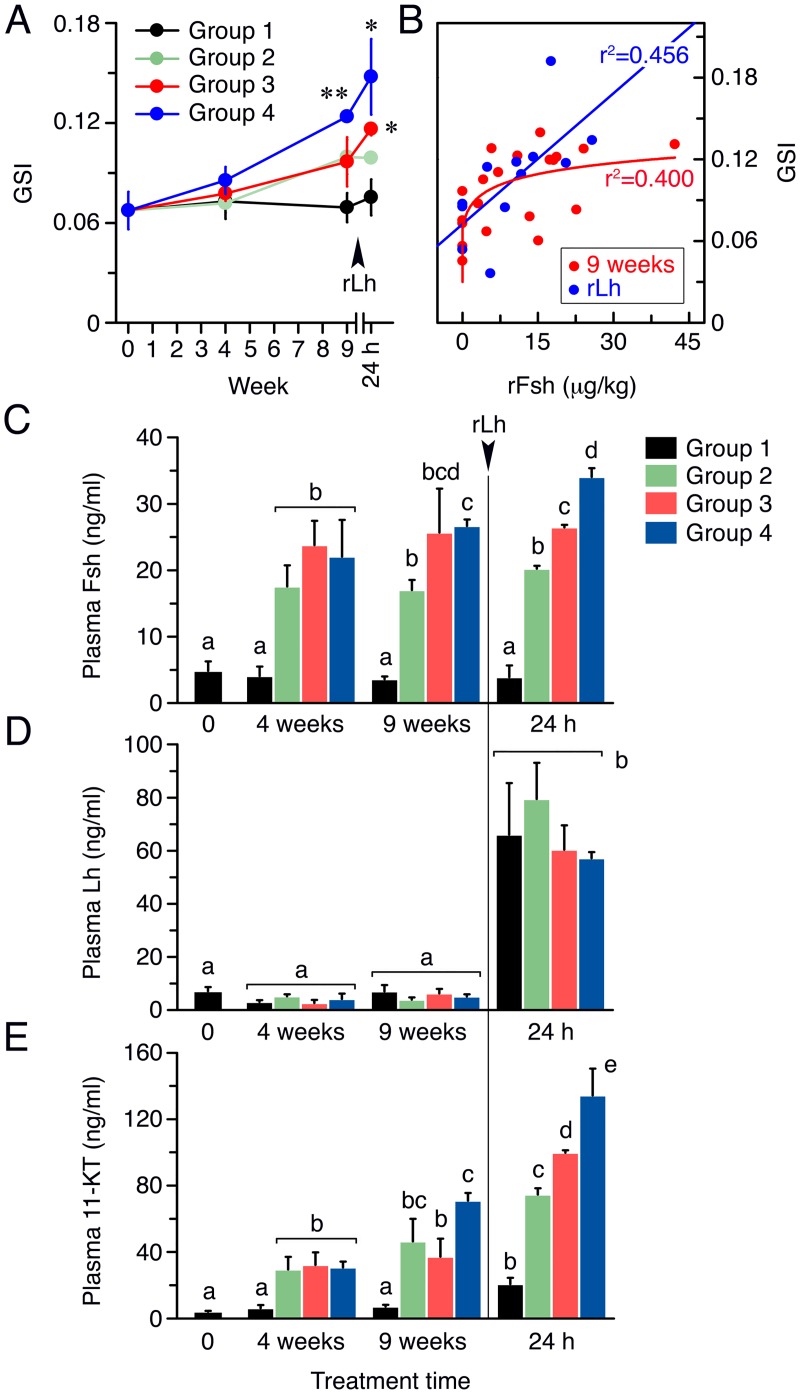
Gonad weight and gonadotropin and androgen circulating levels in males treated with rFsh and rLh in experiment 1. (A) GSI of males from Groups 1 to 4 after 4 and 9 weeks of treatment, and 24 h after the injection with a high dose of rLh. *, *P* < 0.05; **, *P* < 0.01, with respect to the Group 1. (B) Regression analyses of the GSI of each male with the actual dose of rFsh received (in μg/kg) at 9 weeks, and at 24 h after rLh injection. (C-E) Plasma levels of Fsh (C), Lh (D) and 11-KT (E) in each group after 4 and 9 weeks of treatment (measured one week after injection), and 24 h after rLh injection. Bars with different superscript are significantly different (*P* < 0.05). In A and C-E panels, data are the mean ± SEM (*n* = 5 fish, and *n* = 3 fish at 24 h).

Evaluation of the GSI of fish treated with rFsh plus a low rLh dose for 4 and 9 weeks showed that the weight of the testis in Group 4 significantly increased with respect to the saline-injected fish (Group 1), whereas the GSI of Groups 2 and 3, which also increased with the treatment time, were not statistically significant with respect to Group 1 (Fig 3A). However, one day after the last injection with a high rLh dose, the GSI slightly increased in Group 1, while in Groups 3 and 4 it became significantly higher than in Group 1 (Fig 3A). Nonlinear regression analysis of the GSI values for each fish after 9 weeks of treatment and the dose of rFsh per kg injected suggested the existence of a limit in the rFsh dose above which the GSI could not increase further (Fig 3B). In contrast, the best correlation between the GSI and the rFsh doses after the rLh injection was linear, possibly suggesting that the Lh-induced increment of the GSI was dependent on the amount of rFsh administered prior to the rLh treatment (Fig 3B).

After 4 weeks of rFsh plus rLh treatment, plasma levels of Fsh in Groups 2–4 measured one week after injection were significantly higher than in Group 1, although the three rFsh-treated groups showed similar levels (~20 ng/ml) regardless of the dose administered (Fig 3C). However, at 9 weeks the Fsh plasma levels in Group 4 were significantly higher than in Group 2, whereas the concentrations of Fsh in Group 3 were not significantly different from those in Group 2 or 4 (Fig 3C). At 24 h after rLh injection, a dose dependent increase in plasma Fsh was detected, with levels reaching ~34 ng/ml in Group 4 (Fig 3C). Determination of the Lh plasma levels in Groups 1–4 indicated that injection of rFsh did not affect the endogenous concentrations of Lh, which remained <10 ng/ml up to 9 weeks in all groups (Fig 3D). However, one day after rLh injection, the Lh plasma levels increased by ~6 times in the four groups (Fig 3D). As a result of rFsh treatment, the 11-KT plasma levels in Group 2 to 4 were ~6 times higher (~30 ng/ml) than in Group 1 (~5 ng/ml) after 4 weeks, whereas at week 9 a further increase of the androgen concentrations was observed in Groups 2–4, although it was only significant for Group 4 (Fig 3E). The subsequent 24 h treatment with rLh increased the 11-KT plasma levels in Group 1 (~20 ng/ml), and further elevated the androgen concentrations in Groups 2 to 4 in a dose-dependent manner (~70, 90 and 110 ng/ml, respectively) (Fig 3E).

#### rFsh and rLh treatment promote spermatogonial proliferation, meiosis initiation, and spermiogenesis

**Fig 4:**
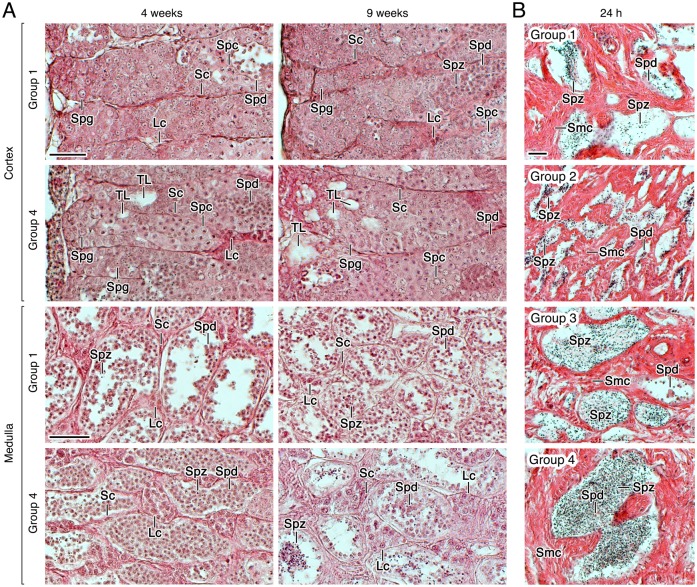
Testicular development of males treated with rFsh and rLh in experiment 1. (A) Representative photomicrographs of histological sections from the cortical and medullar regions of the testis of Group 1 and 4 stained with hematoxylin and eosin after 4 and 9 weeks of treatment. (B) Photomicrographs of histological sections from the testicular sperm duct stained with hematoxylin and eosin from Groups 1 to 4 at 24 h after rLh injection. Scale bars, 20 μm. Sc, Sertoli cell; Lc, Leydig cell; Spg, spermatogonia; Spc, spermatocyte; Spd, spermatid; Spz, spermatozoa; TL, forming tubular lumen.

The histological evaluation of the testis from fish treated with rFsh and a low rLh dose revealed visible changes occurring in the cortical part of the testis, a region that encloses the seminiferous tubules, which often do not have the central lumen formed and usually contain germ cells at all developmental stages (spermatogonia, spermatocytes, and spermatids), but with very few spermatozoa [19]. In Group 1, many spermatogonia were packed within the tubules and some spermatocytes and spermatids could be observed after 4 weeks of treatment (Fig 4A). In fish treated with the highest dose of rFsh (Group 4), the cortical testis contained tubules with a higher number of spermatocytes than in Group 1, and showed the formation of new spermatogenic tubules (Fig 4A). These effects were more pronounced after 9 weeks of rFsh treatment, but, interestingly, one day after the rLh injection, the newly formed tubules seemed to be filled with spermatogonia (Fig 4A). In contrast, very few differences between Groups 1 and 4 were observed in terms of spermatid number within the medullar tubules, although in Group 4 some tubules showed somewhat more spermatozoa than in Group 1 (Fig 4A). Regarding the interstitial cells, the Leydig cell foci delimitated by the spermatogenic tubules typically contained 2–4 cells in Group 1, whereas this number was sometimes increased to >10 in Group 4 (Fig 4A). Finally, the examination of the sperm ducts from Groups 1 to 4 showed a progressive increase in the number of spermatozoa within the duct lumen according to the rFsh treatment (Fig 4B).

**Fig 5:**
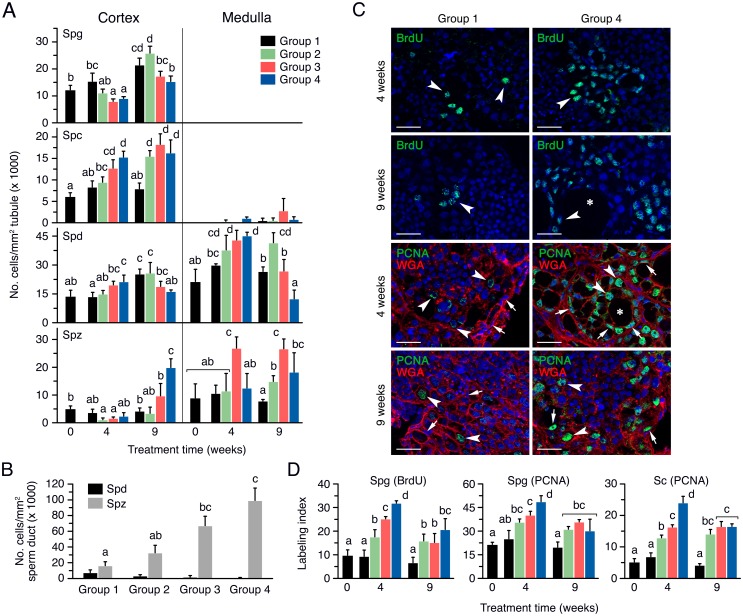
Quantitative assessment of germ cell development in males treated with rFsh and rLh in experiment 1. (A) Number of germ cells normalized by the surface of the tubule in the cortex and medulla of the testis in males from Groups 1 to 4 after 4 and 9 weeks of treatment. (B) Number of spermatids and spermatozoa in the sperm duct, normalized by the surface of the duct lumen, in Groups 1 to 4 at 24 h after rLh injection. (C) Representative BrdU and PCNA immunostaining, counterstained with Alexa Fluor^®^ 488-conjugated WGA, in the testicular cortical region of males from Groups 1 and 4 in A. The arrowheads and arrows indicate proliferating spermatogonia and Sertoli cells, respectively, whereas the asterisks point to the lumen of a newly forming tubule. Scale bars, 20 μm. (D) Percentage of BrdU- and PCNA-positive cortical spermatogonia, and of PCNA-positive Sertoli cells, in the groups shown in C. In panels A, B and D, data are the mean ± SEM (*n* = 5 fish, and *n* = 3 fish in B), and bars with different superscript are significantly different (*P* < 0.05). Spg, spermatogonia; Spc, spermatocyte; Spd, spermatid; Spz, spermatozoa.

To confirm the effects of the rFsh treatments on spermatogenesis, the number of the different types of germ cells in each testicular seminiferous tubule from the cortex and medulla was scored. These values were normalized by the area of the tubules, which did not change among treatments (S1 Fig). In the cortex, the number of spermatogonia per tubule in Group 1 increased at week 9, whereas in Groups 2 to 4 the amount of spermatogonia with respect to the control group tended to progressively decrease, although the differences were statistically significant only for Groups 3 and 4 at 4 weeks and Group 4 at 9 weeks (Fig 5A). The occurrence of spermatocytes in the tubules in Group 1 did not change during the experiment (Fig 5A). However, in Groups 2 to 4, contrary to that observed for spermatogonia, the number of spermatocytes with respect Group 1 was increased at 4 and 9 weeks, the increment showing a dose-response effect with the rFsh dose after 4 weeks of treatment (Fig 5A). The number of spermatids per tubule in the different groups showed the same pattern than that of the spermatocytes at week 4, but at week 9 a progressive decrease of the spermatid number with the rFsh dose was observed, which was associated with a significant increase in the number of spermatozoa in Group 4 (Fig 5A).

In the medullar region of the testis, the tubules contained spermatids and spermatozoa, and a low number of spermatocytes which did not change significantly among the groups (Fig 5A). The number of spermatids in Group 1 remained similar during the experiment, while the rFsh-treated groups showed a progressive increase in the amount of spermatids at week 4 (Fig 5A). In contrast, as observed in the cortex, the population of spermatids gradually decreased at week 9, although it was statistically significant only for Group 4, whereas in Group 2 the spermatid number reached higher levels than in the control group (Fig 5A). The number of spermatozoa in the medulla in Group 3 was higher than in Group 1 at week 4, but the amount of these cells in Groups 2 and 4 was not different than in the control group (Fig 5A). However, at week 9 the number of spermatozoa was higher in the three rFsh-treated groups with respect to the Group 1 (Fig 5A). After rLh injection the number of spermatozoa was strongly increased in a rFsh dose-dependent manner, with Group 4 showing ~6 times more spermatozoa than the controls, which was associated with the progressive decrease in the number of spermatids (Fig 5B).

The pattern of germ cell development in the four experimental groups suggested that rFsh was able to induce the formation of new tubules and the division of spermatogonia and entry into meiosis. To assess the effect of the rFsh injection on spermatogonial proliferation, the number of BrdU-positive cells in the cortical region of the testis pieces previously incubated with the thymidine analog BrdU were scored. The BrdU staining was exclusively observed in the nucleus of spermatogonia (Fig 5C), and no staining was detected in spermatocytes, indicating that short-term incubation of the sole testis with the dye specifically stains mitotic events [46]. Few BrdU-positive spermatogonia were observed in Group 1 after 4 weeks of treatment, while in Group 4 some tubules were entirely filled with dividing cells (Fig 5C). After 9 weeks, similar staining was observed in Group 1 whereas in Group 4 the number of BrdU-positive spermatogonia seemed to increase, especially in areas surrounding the newly forming tubules (Fig 5C). The BrdU labeling index indicated that in Group 1 up to 10% of spermatogonia were dividing throughout the experiment (Fig 5D). In contrast, the rFsh treatment induced a dose-dependent increase in the mitotic index at 4 weeks, reaching ~33% in Group 4, while in Groups 2 and 4 this increase was slightly lower but still reaching significantly higher levels than in the controls after 9 weeks of treatment (Fig 5D).

Since BrdU staining in the previous experiments was not clearly detected in somatic cells, we carried out PCNA immunostaining to investigate further whether the hormone treatments also induced the proliferation of Sertoli cells. These experiments showed that the rFsh treatment stimulated the percentage of PCNA-positive spermatogonia in Groups 2 to 4 at week 4 and 9 (Fig 5C and 5D), thus supporting previous observations with BrdU incubation. The PCNA staining also revealed that the percentage of PCNA-labelled Sertoli cells increased at week 4 and 9 in a rFsh dose-dependent manner (Fig 5C and 5D), thus showing the same pattern than that of the spermatogonia, which confirmed that the rFsh treatments also promoted the proliferation of Sertoli cells.

#### rFsh induction of Leydig cell proliferation

**Fig 6:**
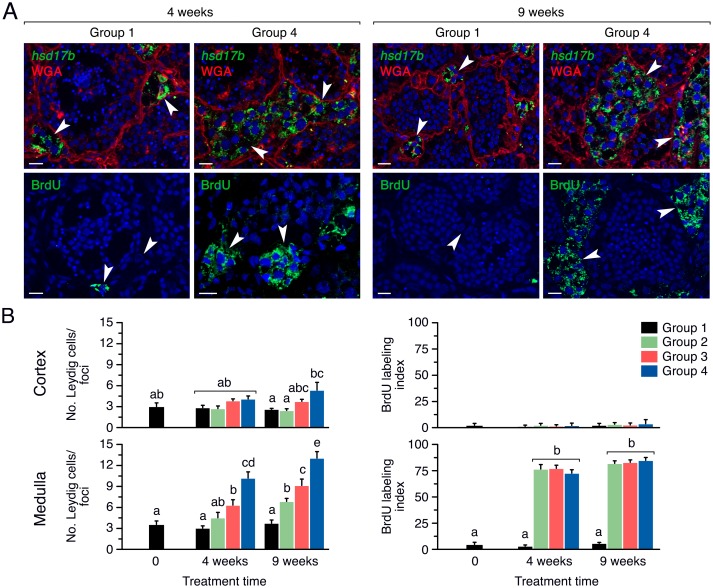
Induction of Leydig cell proliferation by rFsh in experiment 1. (A) Representative *hsd17b in situ* hybridization counterstained with Alexa Fluor^®^ 488-conjugated WGA (upper panels), and BrdU immunostaining (lower panels), in the testicular medullar region of males from Groups 1 and 4 after 4 and 9 weeks of treatment. Sense probes for *hsd17b* were negative (data not shown). Scale bars, 10 μm. (B) Number of Leydig cells per foci in the cortex and medulla of the testis (left panels) and percentage of BrdU-positive Leydig cells (right panels) in Groups 1 to 4 after 4 and 9 weeks of rFsh treatment. Data are the mean ± SEM (*n* = 5 fish), and bars with different superscript are significantly different (*P* < 0.05).

The histological analysis revealed that Leydig cells were more abundant in the medulla of the testis from fish treated with rFsh. The identity of these interstitial cells as Leydig cells was confirmed by FISH labeling with the specific steroidogenic marker *hsd17b* (Fig 6A, upper panels). Further BrdU staining revealed that in Leydig cells the dye, in addition to the nucleus, was mostly observed in the cytoplasm, which could correspond to an active replication of the mitochondrial DNA [47]. The pattern of staining also indicated that the number of BrdU-positive Leydig cells and the size of the foci were clearly incremented in Group 4 with respect the controls (Fig 6A, lower panels). Quantitative assessment of the number of Leydig cells per foci in the cortical and medullar region of the testis showed that in the cortex the amount of cells was slightly increased with the rFsh treatments after 9 weeks, as well as after the rLh induction, but very few of these Leydig cells were BrdU-positive (Fig 6B, upper panels). However, the rFsh treatments strongly promoted the proliferation of Leydig cells in the medulla during the entire experiment, with Group 4 showing 4 times more cells than Group 1 (Fig 6B, lower panels). Accordingly, Groups 2 to 4 exhibited a BrdU labelling index of ~90% while it was only ~5% in Group 1 (Fig 6B, lower panels). These observations were confirmed by using PCNA staining (S2 Fig).

#### Modulation of expression of spermatogenesis-related transcripts by rFsh

**Fig 7:**
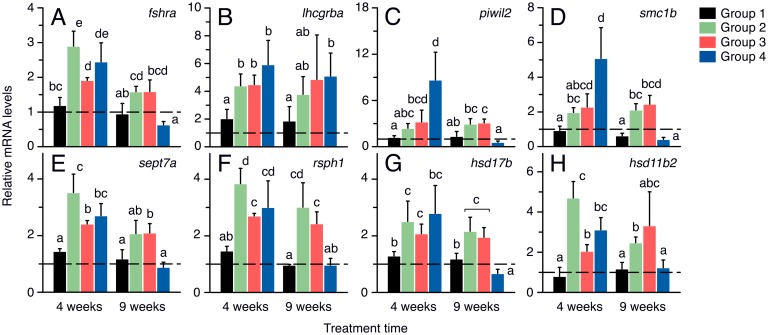
Changes in the expression of testicular genes in males treated with rFsh and rLh in experiment 1. Relative mean expression levels of *fshra*, *lhcgrba*, *piwil2*, *smc1b*, *sept7a*, *rsph1*, *hsd17b* and *hsd11b2*, normalized to *rps18* gene expression, after 4 and 9 weeks of treatment. Values are expressed as fold-changes with respect to time zero. Dashed line at mRNA level 1 indicates no change with respect time zero. Data are the mean ± SEM (*n* = 5 fish), and bars with different superscript are significantly different (*P* < 0.05).

To assess whether the hormone effects on germ cell development were associated with changes in the expression of spermatogenesis-related genes, we performed qRT-PCR analysis of the *fshra* and *lhcgrba*, as well as of several genes related to meiosis, spermiogenesis and steroidogenesis (Fig 7). For these analyses, biopsies of testes enriched in the cortical region were employed. After 4 weeks of treatment, the *fshra* transcripts were upregulated in Groups 2–4 with respect to Group1, while at 9 weeks a tendency to downregulate the *fshra* with the rFsh dose was observed (Fig 7A). Similarly, the *lhcgrba* mRNA was accumulated in the testis of Groups 2 to 4 after 4 weeks of treatment, but in this case the levels of *lhcgrba* tended to remain more elevated than in the controls after 9 weeks (Fig 7B). The two germ cell markers investigated, *piwil2* and *smc1b*, showed a similar pattern of regulation by rFsh, a dose-dependent stimulation at 4 weeks, which was maintained in Groups 2 and 3 at 9 weeks, whereas the longer treatment time resulted in downregulation of both genes in Group 4 (Fig 7C and 7D). The same regulation was observed for the two spermiogenesis markers, *sept7a* and *rsph1* (Fig 7E and 7F), and the androgenic enzymes *hsd17b* and *hsd11b2*, involved in testosterone and 11-KT synthesis (Fig 7G and 7H). The expression of these genes was potentiated by all rFsh doses at 4 weeks, but only by low doses of the hormone after 9 weeks (Fig 7E and 7F).

### Experiment 2: effect of rFsh pretreatment on rLh-induced sperm production

The second experiment, conducted during the natural prespawning and spawning period of Senegalese sole, was aimed at evaluating the effect of one rLh injection (6 μg/fish) on androgen release and sperm production and quality in males previously treated with rFsh (6 μg/fish) for 4, 6 and 8 weeks. In this case, the weekly rFsh treatments were dispensed alone without a low dose of rLh as in experiment 1 to prevent a premature induction of spermiation. Four groups of males were established: Group 5, treated with saline for 9 weeks; Group 6, treated with saline for 9 weeks and also with rLh at week 4, 6 and 8; Group 7, treated with rFsh for 9 weeks and also with saline at week 4, 6 and 8; and Group 8, treated with rFsh for 9 weeks and also with rLh at week 4, 6 and 8. To detect possible negative effects of a prolonged exposure to high hormone doses, the treatments were switched at week 10 and Groups 5 and 7 received a single injection of rLh whereas Groups 6 and 8 were injected with saline.

#### Effect of combinations of rFsh and rLh treatments on androgen plasma levels

**Fig 8:**
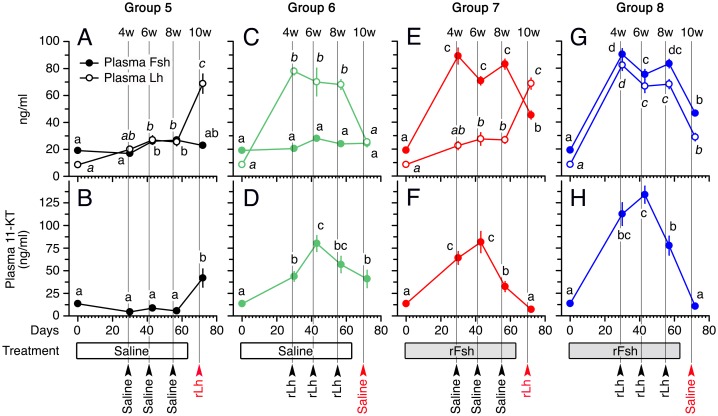
Plasma levels of Fsh, Lh and 11-KT, in males treated with rFsh and rLh in experiment 2. Concentrations of Fsh and Lh (A, C, E and G) and 11-KT (B, D, F and H) in males from Groups 5 to 8 after 24 h (4 weeks) or 48 h (6, 8 and 10 weeks) of the rLh treatment. For each hormone, values with different superscript are significantly different (*P* < 0.05).

Before the experiment, the basal endogenous plasma levels of Fsh and Lh were ~20 ng/ml and ~10 ng/ml, respectively (Fig 8A). In Group 5, the Fsh levels increased slightly but significantly after 6 and 8 weeks, and declined thereafter at week 10 (Fig 8A). A slight increase in plasma Lh was noted at 6 and 8 weeks (~20 ng/ml), and two days after the rLh injection at week 10 the levels reached ~70 ng/ml (Fig 8A). Despite the changes in Fsh and Lh observed in this group during the first 8 weeks, the plasma 11-KT levels remained <12 ng/ml, increasing to ~45 ng/ml after the rLh induction at week 10 as expected (Fig 8B).

Group 6 showed the same seasonal variations of Fsh as Group 5, but in this case the plasma levels of Lh at week 4, 6 and 8 were increased to ~70–80 ng/ml as a consequence of the repeated rLh injections (Fig 8C). However, at week 10, when saline was injected, the Lh concentrations dropped to basal levels (~20 ng/ml) (Fig 8C). In Group 6, the rLh treatments induced a progressive increase of 11-KT at week 4 and 6 (~40 and 75 ng/ml, respectively), but unexpectedly a small decline in the androgen levels was noted at week 8 (~55 ng/ml) after rLh injection, which continued to decrease at week 10 (~40 ng/ml) in the absence of rLh (Fig 8D).

In Group 7, the Fsh plasma levels at 4, 6 and 8 weeks were high (~70–90 ng/ml) as a result of the weekly injection with rFsh, and after the saline injection at week 10 they decreased to ~40 ng/ml (Fig 8E). The plasma levels of Lh until week 8 showed the same pattern as in Group 5, whereas a further increase up to ~70 ng/ml occurred after rLh injection (Fig 8E). Accordingly, the 11-KT plasma levels in this group at 4 and 6 weeks were elevated (~60–75 ng/ml), but despite the presence of high levels of Fsh or Lh during weeks 8 and 10, respectively, the circulating androgen levels dropped strongly to ~35 and 5 ng/ml at these sampling times as observed in Group 6 (Fig 8F).

The same pattern of response to the recombinant hormones was noted in Group 8, since in this group the combined treatment with rFsh and rLh during weeks 4, 6 and 8 resulted in high plasma levels of both hormones, which were comparable to those elicited when the hormones were applied separately in the other groups (Fig 8G). Treatment of these groups with rFsh and rLh during weeks 4 and 6 increased the circulating 11-KT to higher levels (110–130 ng/ml) than in the other groups injected separately with either of the hormones, confirming the steroidogenic potency of both recombinant hormones (Fig 8H). However, as in Group 7, rLh treatment at week 8 decreased the 11-KT plasma levels to ~75 ng/ml, which reached ~5 ng/ml after saline injection at week 10 (Fig 8H).

#### Viability of Leydig cells exposed to rFsh and rLh

**Fig 9:**
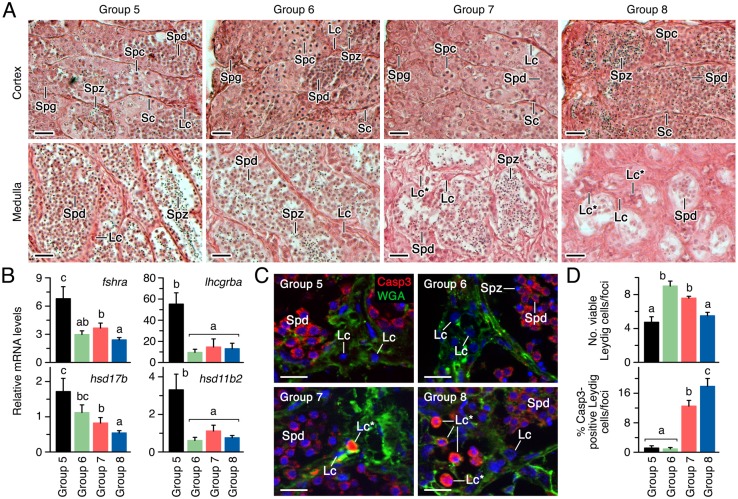
Effect of rFsh and rLh treatments on testicular development and Leydig cell survival in experiment 2. (A) Representative photomicrographs of histological sections stained with hematoxylin and eosin from the cortical and medullar regions of the testis of males from Groups 5 to 8 after the completion of the experiment at week 10. (B) Expression levels of *fshra*, *lhcgrba*, *hsd17b* and *hsd11b2* in the testis at the same time normalized to *rps18* gene expression. (C) Representative immunostaining of Casp3 counterstained with WGA in the testicular medullar region of males. (D) Number of live (upper panel) and Casp3-expressing (lower panel) Leydig cells in the medullar region of the testis. In A and C, scale bars, 10 μm. In B and D, data are the mean ± SEM (*n* = 5 fish), and bars with different superscript are significantly different (*P* < 0.05). Lc, Leydig cell; Lc*, Casp3 expressing (apoptotic) Leydig cell; Spd, spermatid; Spz, spermatozoa.

To investigate the causes of the low steroidogenic response of fish continuously treated with the recombinant hormones, we first carried out histological analyses of the testes of a subsample of fish in each experimental group after 10 weeks of treatment. These analyses revealed that the interstitial Leydig cells present in the testicular cortical region showed no apparent differences between the four groups (Fig 9A). However, the occurrence of spermatocytes in the cortical tubules was clearly lower in Group 8 with respect to the other groups, while in this group spermatozoa entirely filled some of the tubules (Fig 9A). In contrast, the spermatids and spermatozoa were often absent from the medullar tubules of Group 8, and to a lesser extent also in Group 7 (Fig 9A). Nevertheless, the most striking observation was on the medullar interstitial regions of testis from Groups 7 and 8, which appeared more fibrous and contained foci of Leydig cells encompassing a relatively large number of abnormal appearing cells, unlike that observed in the cortex (Fig 9A).

The evaluation of the expression levels of various markers of Leydig cell function in the testis indicated that *fshra*, *lhcgrba*, and *hsd11b2* were significantly down regulated in Groups 6 to 8, whereas the *hsd1b7* transcripts were only significantly reduced in Groups 7 and 8 (Fig 9B). To investigate the presence of apoptotic Leydig cells in Groups 6 to 8, which could explain the reduced testicular steroidogenesis, immunostaining for the apoptosis marker cleaved Casp3 was performed. Interestingly, Casp3 was strongly expressed in the cytoplasm of released spermatids within the medullar tubules (Fig 9C), which supports previous findings in mammals indicating that spermatid individualization, a process unrelated to the cell death pathway [48], is dependent of Casp3 activation [49]. The Casp3 antibody also strongly stained the degenerative nucleus of many of the Leydig cells from Groups 7 and 8 (Fig 9C). Quantification of live and apoptotic Leydig cells in the medulla of the testis from each group indicated that the number of viable cells per foci was higher in Groups 6 and 7 than in Group 5, and significantly reduced in Group 8, whereas the percentage of Casp3-positive cells significantly increased by 10% and 16% in Groups 7 and 8, respectively (Fig 9D). These data therefore suggested that the continuous treatment of male fish with rFsh induced apoptosis of Leydig cells.

#### Differential induction of sperm production by gonadotropin treatments

**Fig 10:**
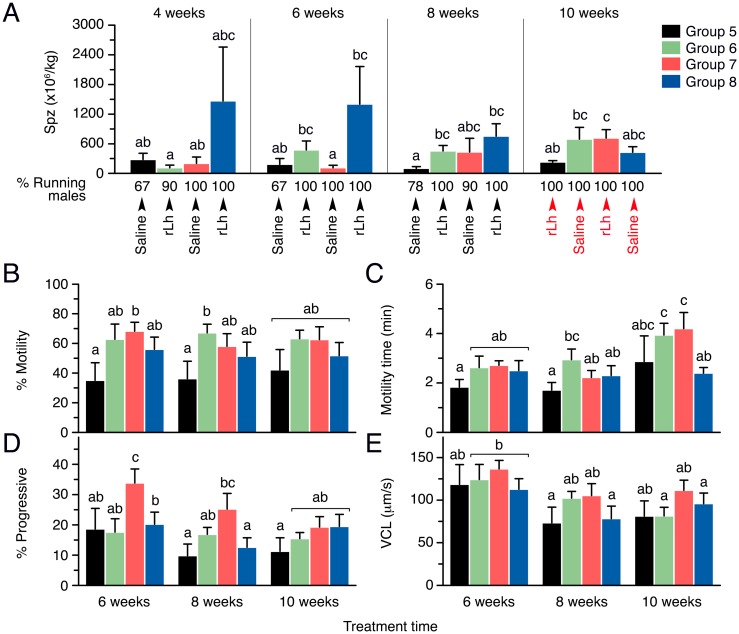
Sperm production and kinetic parameters of sperm by males treated with rFsh and rLh in experiment 2. (A) Mean amount of sperm produced by each group after saline or rLh injection during weeks 4, 6, 8 and 10. Below each panels is indicated the treatment received and the percentage of running (spermiating) at each time point. (B-E) Kinetic parameters of the sperm produced during each week. In all panels, the data are the mean ± SEM (*n* = 10 fish), and bars with different superscript are significantly different (*P* < 0.05).

The effects of the rFsh treatment on the production and kinetics of sperm were assessed one (at 4 weeks) or two (at 6, 8, and 10 weeks) days after the rLh injection (Fig 10). The percentage of spermiating males in Group 5 was <80% at weeks 4, 6 and 8, and only increased to 100% following the rLh injection after 10 weeks, whereas in the other groups almost all males (>90%) were spermiating during all sampling times (Fig 10A). After the first rLh injection at 4 weeks, the production of sperm in Groups 5 to 7 was of 281 and 201 x 10^6^ spermatozoa/kg, respectively, whereas the males from Group 8 produced a mean of 1,400 x 10^6^ spermatozoa/kg (Fig 10A). However, in this latter group sperm production between males was highly variable ranging from 55 to >1500 x 10^6^ spermatozoa/kg in 30% of the fish, and consequentially the group mean with a high standard deviation was not statistically significantly different from the other groups. At 6 weeks, Group 6 showed a tendency to produce more spermatozoa than Group 5 and 7 in response to rLh, whereas the production by Group 8 remained similar than that at week 4, but became ~12 times higher than in Group 7 (Fig 10A). A similar response was observed when fish were injected with rLh after 8 weeks of treatment, although in this case Group 7 also increased the production of sperm, whereas Group 8 started to decrease the amount of spermatozoa emitted (Fig 10A). At this time, however, the variability in the response to rLh within each group was lower, and the data indicated that the number of spermatozoa produced by Groups 6 and 8 was ~4 and ~7 times significantly higher, respectively, than in Group 5 (Fig 10A). At 10 weeks, when the treatment with rLh was switched between the groups, both Groups 5 and 7 receiving rLh tended to raise the sperm produced with respect to that observed after saline injection at week 8, the production however being ~3 times higher in Group 7 pretreated with rFsh than in Group 5. For Groups 6 and 8 receiving a saline injection at this time the production of sperm tend to increase and decrease, respectively, with respect to that noted in these groups at week 8 (Fig 10A).

During the weeks 6, 8 and 10, different sperm kinetic parameters were evaluated by CASA. Group 5 showed a mean percentage of motile sperm of ~30–40% at the three sampling times, whereas sperm motility tended to increase up to ~70% in the hormone-treated Groups 6 to 8 (Fig 10B). The time of motility of spermatozoa also showed a tendency to be higher in Groups 6 to 8 at week 6 and 8, although the differences with respect Group 5 were not significant at all sampling times (Fig 10C). At week 10, all groups also tended to show longer motility times, except for Group 8, which produced less motile spermatozoa than those produced by Groups 6 and 7 (Fig 10C). Similarly, few differences among groups were seen in the progressivity of spermatozoa, except that in Group 7 it was enhanced with respect to Group 5 at 6 and 8 weeks, but not at 10 weeks (Fig 10D). Finally, the VCL did not change significantly among the groups during any of the sampling times (Fig 10E).

## Discussion

The present findings demonstrate that hormone therapies using homologous recombinant gonadotropins can effectively promote spermatogenesis and improve sperm production in Senegalese sole males raised in captivity.

Teleost recombinant gonadotropins produced in mammalian CHO cells have previously been shown to be more stable, and possibly more bioactive *in vivo*, than hormones produced in other heterologous systems [38]. This is likely due to the ability of mammalian cells to express glycoproteins with complex sialylated oligosaccharides [38, 50]. Accordingly, in experiment 1 of this study both rFsh and rLh stimulated the testicular expression of the steroidogenic enzymes *hsd17b* and *hsd11b2* and greatly increased androgen release *in vivo*, which plasma levels were still higher than the controls one week after injection. After 24 h of rLh treatment, the 11-KT levels increased in a rFsh dose-dependent manner, which were correlated with a progressive rFsh-induced proliferation of Leydig cells particularly in the medulla of the testis, suggesting that the testicular steroidogenic potency was determined by rFsh. These observations are in accordance with the role of Fsh in Leydig cell proliferation described in lizards [51], or with the fact that FSHR and FSHβ null mice exhibit lower Leydig cell proliferation rates [52, 53]. However, because the Fshra is expressed in Leydig cells in Senegalese sole [18], it remains unknown whether the effect of rFsh on the proliferation of these somatic cells occurs directly through the activation of its cognate receptor, or indirectly via rFsh-stimulated release of mitogenic factors by Sertoli cells.

In experiment 2, when rFsh and rLh were injected together, the effect on circulating 11-KT was highly increased, suggesting an enhanced steroidogenic response by the Leydig cells expressing both the Fsrha and Lhcgrba. In this experiment, however, the 11-KT levels increased in response to rFsh and rLh during the first 6 weeks, but longer treatment times with the hormones reduced or completely abolished the testis androgen responsiveness to rFsh or rLh. Similar results have recently been reported in other teleosts such as the yellowtail kingfish (*Seriola lalandi*), where 11-KT is reduced by homologous rFsh in early pubertal males [54], and the Russian sturgeon (*Acipenser gueldenstaedtii*), in which 11-KT secretion *in vitro* by prepubertal testis is decreased by high doses of rFsh while low doses were highly effective [55]. In our study, the low androgenic response to gonadotropins of the testis after 8 and 10 weeks of hormone treatment, particularly of rFsh, can be explained by the downregulation of *fshra*, *lhcgrba*, *hsd17b* and *hsd11b2*, which could negatively affect 11-KT synthesis, and by the accumulation of the apoptosis executor cleaved Casp3 in medullar Leydig cells. The potential modulation of Leydig cell survival by Fsh in sole is interesting since in mammals, in which Leydig cells express only the LHCGR, FSH has been described to play an anti-apoptotic role on germ cells but not in Leydig cells [56, 57]. However, in experiment 1 the males treated with the highest rFsh dose were still competent to respond to rLh by secreting 11-KT after 9 weeks, even though the testicular expression levels of *fshra*, *hsd17b* and *hsd11b2* were depressed, although in this case no apoptosis of Leydig cells was noted. These observations suggest that under relative low temperatures in winter (~13°C) sustained rFsh treatment can modulate the expression of steroidogenic enzymes, possibly through a negative feedback mechanism of androgens [58, 59], but does not impair androgen synthesis because high levels of the enzymes may still be present in the Leydig cells. However, when the temperature increases during the spring (~18°C) high circulating levels of rFsh may become toxic to Leydig cells triggering cell death and dramatically reducing the population of steroidogenic cells in the medullar region of the testis. Although the molecular mechanisms underlying these effects require further investigation, the observation that males on an every two week rLh treatment did not show a significant reduction of *hsd17b* expression in testis, or lower Leydig cell survival, supports the hypothesis that they may be Fsh specific.

Senegalese sole males have a small testis (GSI of ~0.06) in which spermatogenesis occurs all-year around, with a slight increase of the GSI (~0.12) and the production of spermatozoa in spring [15, 43]. Treatment of males with rFsh in experiment 1 induced a 2 times increase in the GSI with the highest dose, thus strikingly similar to that observed in naturally maturing males. The analysis of the GSI of each fish after the dose of rFsh actually received per kg suggested that the Senegalese sole testis probably has a physiological limit above which it can not grow regardless of the rFsh dose administered. This observation, although it should be confirmed and investigated in more detail in the future, may be related to the asynchronous type of spermatogenesis of the sole testis. Nevertheless, we also observed that after 24 h of rLh treatment the GSI further increased accordingly with the rFsh dose, possibly due to the rapid hydration of the testis and/or sperm duct [3, 60].

The histological analysis of the testis of the fish treated with rFsh in experiment 1 did not reveal any apparent abnormal or apoptotic event in somatic or germ cells. However, the formation of new tubules was observed in the cortical area of the testis of males treated with the highest rFsh dose during 9 weeks. In mammals, the vacuolization in Sertoli cells seem to precede the formation of the tubular lumen, which is associated with an increase in the number of germ cells [61, 62]. In teleosts, Fsh ensures the survival of spermatogonia and stimulates their proliferation [63, 64], indirectly through the release of Sertoli cells factors such as Insulin-like growth factor 1 or 3 [65, 66], or trypsinogens [67], or through steroids such as progestins [68], 11-KT [69] and estrogens [70]. Accordingly, in the present study spermatogonia and Sertoli cell proliferation was rFsh dose-dependent after 4 weeks of treatment, which agrees with the fact that rFsh is able to promote the upregulation of multiple genes in Senegalese sole Sertoli and Leydig cells *in vitro* [19].

The rFsh-induced proliferation of spermatogonia was followed by the increase of the number of spermatocytes at 4 and 9 weeks, and of spermatids at week 4, after hormone treatment. This observation suggest that rFsh stimulated the initiation of meiosis, since the total number spermatogonia per tubule tended to decrease at the same time periods. In different teleosts, Fsh has been associated with the entry into meiosis during spermatogenesis [63], and thus meiosis-related genes such as *piwil2* and *scmb1* [19, 64] were upregulated in the testis after 4 weeks of rFsh treatment. However, the expression of these genes tended to decrease to the levels in the controls after 9 weeks, probably because spermatocytes differentiate to spermatids, as indicated by the upregulation of spermatid-specific markers such as *sept7a* and *rshp1* [19]. As expected, the spermatid number in the medullar tubules increased after rFsh treatment, and following the subsequent rLh injection the sperm duct was filled with spermatozoa in a dose-dependent manner according to the rFsh dose previously administered. These observations resemble those previously reported in Senegalese sole treated with hCG, which show the enlargement of the lumen of the sperm ducts, together with an increased amount of spermatozoa [24]. Our data therefore suggest that rFsh induced the accumulation of ‘competent’ haploid spermatids in the medulla, which could rapidly (~24 h) differentiate to spermatozoa in response to the rLh, possibly through the activation of the Lhcgrba present in the surface of these cells [20], being subsequently released into the sperm duct.

In the present study, treatment of males with rFsh and rLh, or with rLh alone, increased the number of running (spermiating) males to >90% after only 4 weeks of treatment, whereas the controls showed 67% at the same time, indicating that the recombinant hormones were very effective at stimulating spermiation. Previous studies in Senegalese sole have reported that wild adult males (1.2–1.8 kg) show a variable sperm production from ~50 to 300 x 10^6^ spermatozoa/kg [10, 71]. For F1 males with a similar weight (0.8–1.3 kg) the production of spermatoza has been reported to be from 18 to 100 x 10^6^ spermatozoa/kg [6, 7, 23, 71], whereas treatments with GnRHa alone, or in combination with 11-KT precursors [7] or dopaminergic inhibitors [23], can enhance by ~2 times the total sperm production. In this work, we employed smaller F1 males (~0.3 kg) than in these previous studies, which showed a basal sperm production (with no hormone treatment) slightly higher, of ~280 to 100 x 10^6^ spermatozoa/kg from week 4 to 8, possibly due to differences in the procedures employed for sperm extraction and cell density calculation. Interestingly, treatment with a single rLh injection in spring (week 10) tended to increase the production of sperm by ~2 times, thus to a very similar extent to that previously obtained using GnRHa-based treatments. However, we observed that repeated rLh injections for 8 weeks increased sperm production up to ~4 times, whereas a rLh injection of males previously treated with rFsh for the same time could enhance sperm production up to ~7 times. The positive effect of the hormone treatments on spermatozoa production was also associated to some improvement of their motility, although the sperm motion parameters recorded in the gonadotropin-treated males were in the same range as those reported in previous studies [10, 11, 45, 71, 72]. Nevertheless, our data indicate that the combination of rFsh and rLh treatments was able to stimulate spermatozoa production in cultured Senegalese sole. However, we also observed a high variability in the response of males treated simultaneously with rFsh and rLh, despite that all the fish from this group were spermiating. These phenomena might be related, respectively, to the relative early age of the males used in this study, and the possible toxic effect of the sustained rFsh treatment under the relatively high temperatures of spring.

In summary, the results of the present study show that the rFsh delivery *in vivo* in the Senegalese sole can stimulate gonad growth and the first stages of spermatogenesis, including spermatogonia proliferation and entry into meiosis, whereas the subsequent acute application of rLh potentiates spermatozoa differentiation and their accumulation in the testicular ducts. Accordingly, rLh treatment of rFsh-primed males during prespawning was effective at promoting sperm production, although with high variability among individuals. These data thus suggest that combined treatments with homologous recombinant gonadotropins might be employed as potential hormone therapies to ameliorate reproductive dysfunctions of cultured Senegalese sole males. However, we have also observed that the sustained administration of high amounts of gonadotropins during spawning can become harmful to the testis by affecting Leydig cell survival and their steroidogenic function, which would ultimately reduce sperm production. Further studies are therefore still required to establish the most effective conditions for the induction of sperm production in Senegalese sole by means of recombinant gonadotropins.

## Supporting information

S1 Fig Area of the seminiferous tubules in the testis of males treated with rFsh and rLh in experiment 1. The area of the tubules was estimated by using the NIS-element AR imaging software in the cortex and medulla of the testis in males from Groups 1 to 4. The data are the mean ± SEM (*n* = 5 fish). Values were not statistically different.
(TIF)

S2 Fig Leydig cell proliferation in experiment 1 evaluated by PCNA immunostaining. (A) Representative PCNA detection of cellular proliferation counterstained with Alexa Fluor^®^ 488-conjugated WGA in the testicular medullar region of males from Groups 1 and 4 after 4 and 9 weeks of treatment. Arrowheads indicate interstitial Leydig cells. Scale bars, 10 μm. (B) Percentage of PCNA-positive Leydig cells in the cortex (left panel) and medulla (right panel) at 4 and 9 weeks of treatment. Data are the mean ± SEM (*n* = 5 fish), and bars with different superscript are significantly different (*P* < 0.05).
(TIF)
