# Supplementary figures and images for: Toward developing recombinant gonadotropin-based hormone therapies for increasing fertility in the flatfish Senegalese sole
Source: PLoS One. 2017 Mar 22;12(3):e0174387. doi: 10.1371/journal.pone.0174387 (PMC5362233; doi:10.1371/journal.pone.0174387)

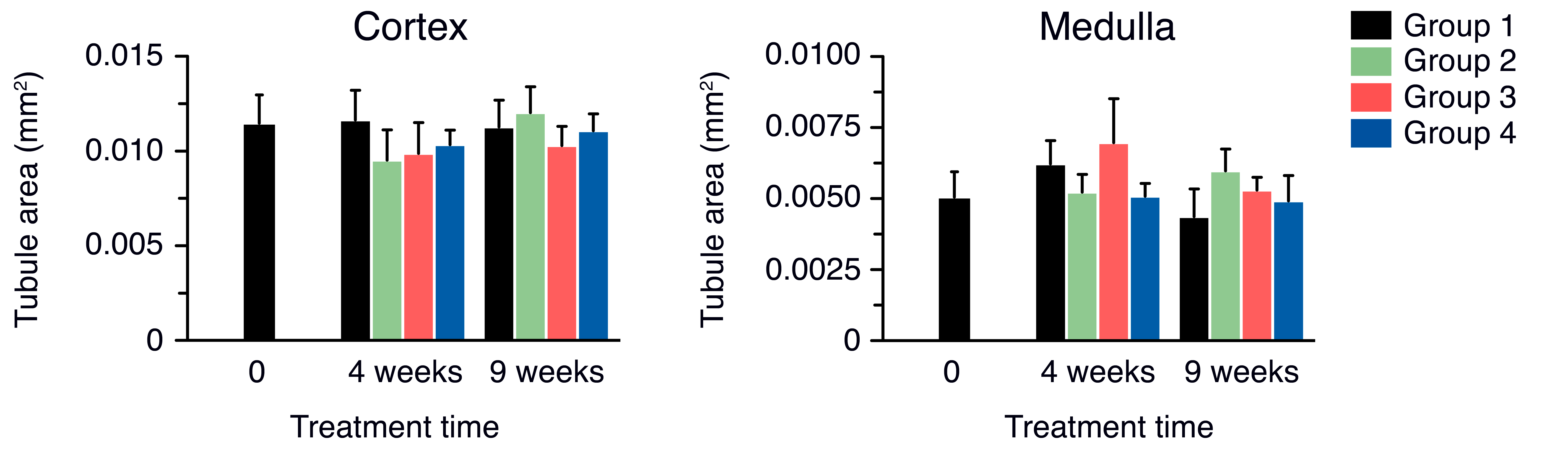

Supplement: S1 Fig — The area of the tubules was estimated by using the NIS-element AR imaging software in the cortex and medulla of the testis in males from Groups 1 to 4. The data are the mean ± SEM (n = 5 fish). Values were not statistically different. (TIF) [file pone.0174387.s001.tif]

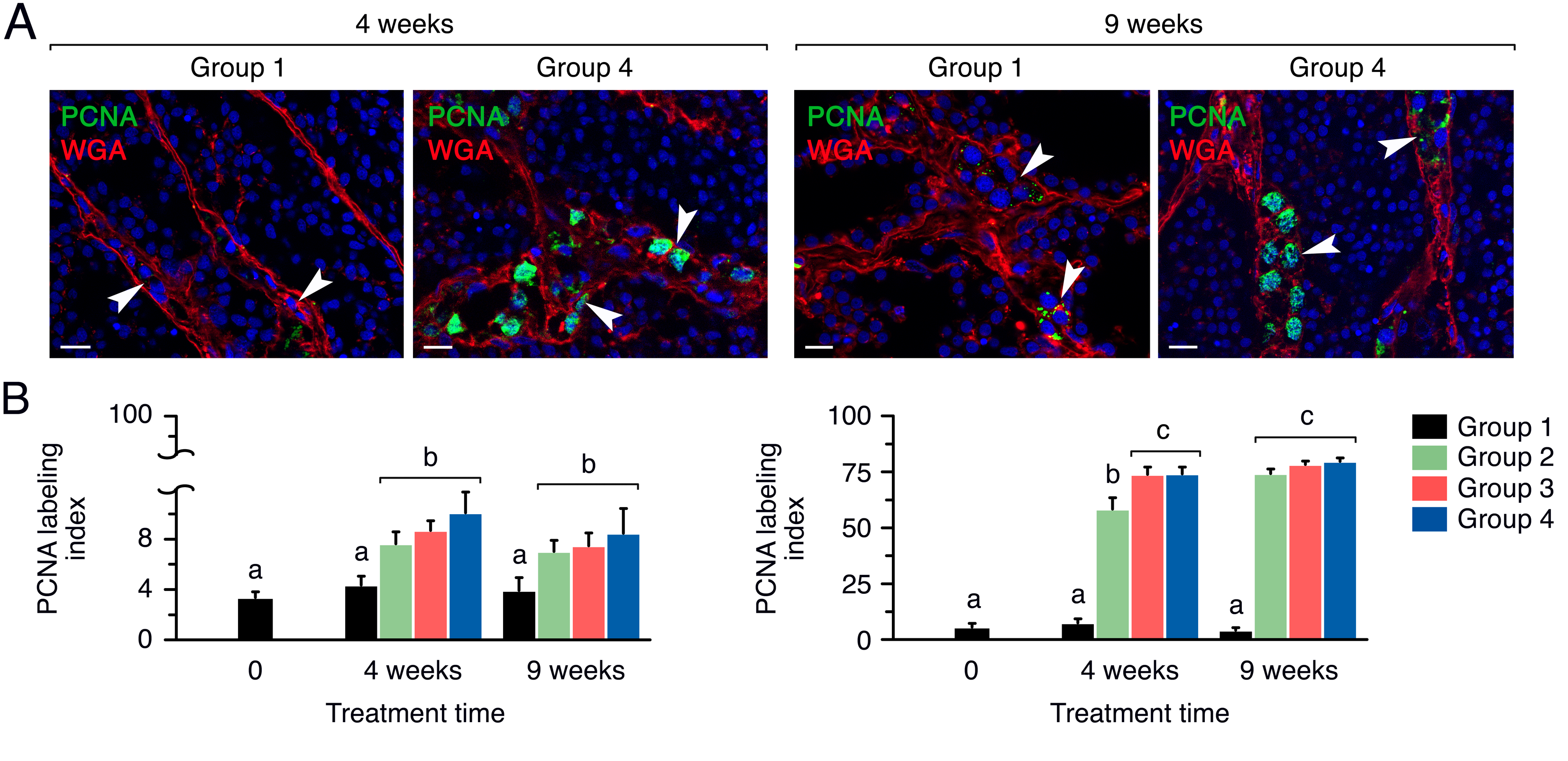

Supplement: S2 Fig — (A) Representative PCNA detection of cellular proliferation counterstained with Alexa Fluor® 488-conjugated WGA in the testicular medullar region of males from Groups 1 and 4 after 4 and 9 weeks of treatment. Arrowheads indicate interstitial Leydig cells. Scale bars, 10 μm. (B) Percentage of PCNA-positive Leydig cells in the cortex (left panel) and medulla (right panel) at 4 and 9 weeks of treatment. Data are the mean ± SEM (n = 5 fish), and bars with different superscript are significantly different (P < 0.05). (TIF) [file pone.0174387.s002.tif]
